# Comparison of Culture, Antigen Test, and Polymerase Chain Reaction for Pneumococcal Detection in Cerebrospinal Fluid of Children

**DOI:** 10.1093/infdis/jiab073

**Published:** 2021-09-01

**Authors:** Md Hasanuzzaman, Senjuti Saha, Roly Malaker, Hafizur Rahman, Mohammad S I Sajib, Rajib C Das, Maksuda Islam, Davidson H Hamer, Gary L Darmstadt, Samir K Saha

**Affiliations:** 1 Child Health Research Foundation, Dhaka, Bangladesh; 2 Microbiology Program, Department of Mathematics and Natural Sciences, Brac University, Dhaka, Bangladesh; 3 Department of Global Health, Boston University School of Public Health, Boston, Massachusetts, USA; 4 Section of Infectious Disease, Department of Medicine, Boston University School of Medicine, Boston, Massachusetts, USA; 5 National Emerging Infectious Disease Laboratory, Boston University, Boston, Massachusetts, USA; 6 Department of Pediatrics, Stanford University School of Medicine, Stanford, California, USA; 7 Dhaka Shishu (Children) Hospital, Dhaka, Bangladesh; 8 Bangladesh Institute of Child Health, Dhaka, Bangladesh

**Keywords:** pneumococcus, diagnostic, meningitis, pneumococcal vaccine, surveillance, antibiotic exposure, vaccine preventable, ICT

## Abstract

**Background:**

Sensitivity of culture for the detection of *Streptococcus pneumoniae* is limited by prior antibiotic exposure. Immunochromatographic test (ICT) is highly sensitive and specific for pneumococcal antigen detection in the cerebrospinal fluid (CSF) of meningitis cases. We determined the specificity and sensitivity of culture, ICT, and polymerase chain reaction (PCR) and the effect of antibiotic exposure on their performance.

**Methods:**

CSF specimens from suspected meningitis cases admitted to Dhaka Shishu Hospital, Bangladesh, were tested using culture, ICT and PCR. Additionally, 165 specimens collected from 69 pneumococcal cases after antibiotic treatment were tested.

**Results:**

Of 1883 specimens tested, culture detected 9, quantitative PCR (qPCR) detected 184, and ICT detected 207 pneumococcal cases (including all culture and qPCR positives). In comparison to ICT, sensitivity of culture was 4.4% and of qPCR was 90.6%; both were 100% specific. After antibiotic exposure, culture sensitivity plummeted rapidly; conventional PCR and qPCR sensitivity disappeared after day 6 and 20, respectively. ICT detected pneumococcal antigen for >10 weeks.

**Conclusions:**

While culture provides the most information about bacterial characteristics, in high antibiotic exposure settings, ICT exhibits maximum sensitivity. We recommend culture and ICT as mainstay for pneumococcal diagnosis and surveillance; qPCR can generate additional molecular data where possible.

*Streptococcus pneumoniae* (pneumococcus) is a leading cause of pneumonia, sepsis, and meningitis in children [1]. In 2015, it was estimated to cause 3.7 million episodes of severe illnesses and 294 000 deaths globally [2]. A large proportion of this burden occurs in low- and middle-income countries. Introduction of 2 conjugate vaccines (PCV-10 and PCV-13) in routine immunization programs has led to significant reduction of invasive pneumococcal diseases in many countries [2–5]. However, these vaccines cover 10 (PCV-10) or 13 (PCV-13) of the 94 known serotypes of pneumococcus. Bangladesh introduced PCV-10 in its routine immunization program in 2015. Previous studies from Bangladesh have reported simultaneous circulation of about 50 invasive serotypes in the prevaccine era, leading to estimated low serotype coverage of the vaccine [6]; this is likely the case in other countries of the region also. With advancing use of pneumococcal vaccines, surveillance data on burden and diversity of pneumococcal disease are imperative in such settings.

Accurate detection and adequate characterization of pneumococcus are key to pneumococcal disease surveillance, but are hampered by a multitude of factors, especially in resource-constrained settings. A large proportion of pneumococcal disease is pneumonia and as most pneumonia cases are nonbacteremic, microbiological diagnosis of pneumococcal cases is specifically difficult [7]. Therefore, clinical and epidemiological studies of serious pneumococcal diseases primarily rely on culture of *S. pneumoniae* from blood or cerebrospinal fluid (CSF) samples [6, 8]. In general, detection of pneumococci by culture can be limited by low blood volume, contamination, and technical expertise. In addition, sensitivity of pneumococcal culture is severely limited by exposure to antibiotics prior to seeking care. A large majority of all cases in South Asia seek care after antibiotic exposure or are administered antibiotics at the hospital before blood collection or lumbar puncture [6, 8–10]. This leaves utilization of more sensitive and culture-independent methods, namely detection of pneumococcal antigen using immunochromatographic tests (ICT) or pneumococcal DNA using conventional or quantitative polymerase chain reaction (cPCR or qPCR, respectively), as the 2 main modes of detection. These tests do not work well for blood samples but have been optimized for CSF samples from pneumococcal meningitis cases. A rapid ICT can detect pneumococcal antigen in the CSF within 15 minutes, making it useful for immediate patient care. However, the currently available rapid test kit (BinaxNOW) is expensive, and is not able to distinguish between serotypes, impeding downstream epidemiological characterization. PCR assays can both detect pneumococcus and decipher serotypes in most cases, but the technique is more time and resource intensive, requires technical expertise, and is not widely available in resource-constrained settings. ICT cannot determine the antibiotic susceptibility patterns of pneumococcus; PCR can successfully predict susceptibility of some antibiotics [11, 12] but culture remains the mainstay for antibiotic susceptibility testing of pneumococcus.

Several previous studies have shown that sensitivity and specificity of ICT is >95% [9, 10, 13]. However, no systematic, head-to-head comparison has been conducted to determine the impact of duration of illness and prior antibiotic exposure on culture, ICT, and PCR to inform evidence-based decisions on what test (or combination of tests) to use when, where, and how. In this study, we aimed to assess the sensitivity and specificity of each of these diagnostics in detection of pneumococcus in Bangladesh, a high-antibiotic–exposure setting, and elucidate the effect of duration of antibiotic exposure on their sensitivity of pneumococcal detection.

## METHODS

### Study Site and Case Selection

**Figure 1.**
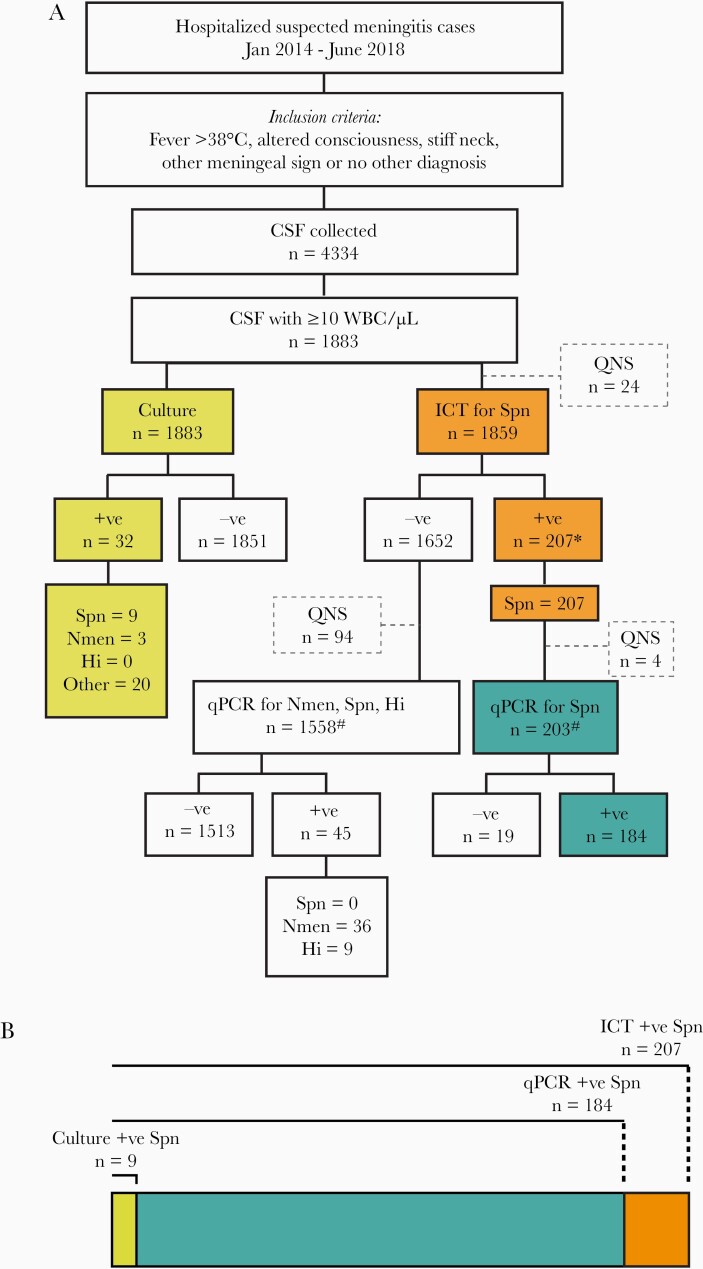
Selection, characteristics, and pathogens detected in the cerebrospinal fluid samples used in the study. *A*, Flow diagram of the diagnostic tests used in the study and results attained. *B*, The total number of pneumococcus detected using each method. *These positives also include the 9 samples detected by culture. ^#^The total of 1558 and 203 samples (1761) included in specificity and sensitivity calculations. Abbreviations: CSF, cerebrospinal fluid; Hi, *Haemophilus influenzae*; ICT, immunochromatographic test; Nmen, *Neisseria meningitidis*; QNS, quantity not sufficient; qPCR, quantitative polymerase chain reaction; Spn, *Streptococcus pneumoniae.*

All CSF samples used in this study were collected as part of the Invasive Bacterial Vaccine Preventable Disease surveillance study supported by the World Health Organization (WHO) conducted at the Dhaka Shishu Hospital (DSH) [14]. DSH, the largest pediatric hospital in Bangladesh, provides primary to tertiary care to children aged 0–18 years. Children admitted to DSH between January 2014 and June 2018 were enrolled if they met the WHO-defined meningitis case definition and they had a CSF specimen collected (Figure 1) [15]. Physicians at DSH advise lumbar puncture from almost all suspected cases of meningitis. In addition, longitudinal CSF samples (>1 CSF samples/case, separated by time, during the same disease episode) are collected from cases, as decrease of baseline white blood cell count and clinical improvement upon antibiotic therapy are considered signs of improvement.

### Laboratory Methods

All tests were performed in the DSH microbiology laboratory of the Child Health Research Foundation, which is a high-performing WHO sentinel site [14]. The laboratory has routinely participated in external proficiency testing programs conducted by the United Kingdom National External Quality Assessment Service, coordinated by WHO, since 2011.

#### Etiology Detection

All CSF specimens were cultured on blood, chocolate, and MacConkey agar plates for bacterial growth. Pneumococcal antigen testing was performed on CSF specimens with ≥10 white blood cells (WBC)/μL using BinaxNow [10]. All subsequent longitudinal CSF specimens from pneumococcus-confirmed cases were simultaneously tested by culture, ICT, qPCR, and cPCR (targeting the *cpsA* gene of pneumococcus [16]), irrespective of the WBC count. Culture-negative and ICT-negative CSF specimens were subjected to multiplex qPCR for *S. pneumoniae* (*lytA* gene), *Haemophilus influenzae* (*hpd* gene), and *Neisseria meningitidis* (*sodC* gene) using protocols described elsewhere [17]. In addition, ICT-positive CSF specimens were tested by qPCR (*lytA* gene).

#### Antibiotic Assay

CSF samples with ≥10 WBC/μL were tested for the presence of antibiotic following a method described earlier [10]. In brief, a blank disc (product CT998; Oxoid) was placed on a lawn of the pan-susceptible organism *Micrococcus luteus* (American Type Culture Collection 9341). A 10-μL CSF sample was pipetted directly on the disc before incubating the plate overnight at 37°C. Any zone of inhibition around the disc was considered positive for the presence of an antibiotic capable of crossing the blood-brain barrier.

### Data Collection and Statistical Analysis

Data on prior antibiotic exposure and antibiotic treatment after hospitalization were recorded for each patient by study physicians. We attempted to collect the exact start date of antibiotic treatment for all pneumococcal-meningitis cases, and from whom longitudinal samples were collected during their hospital stay. Of the 69 pneumococcal cases that provided longitudinal samples, the exact date could be determined for 30 cases, while for the remaining 39 cases, the exact date could not be recalled by the caregivers. For CSF samples that were positive for the presence of an antibiotic (determined by the antibiotic assay described above) but for which the exact start date of antibiotic treatment was not available, we extrapolated the start date based on the cycle threshold (Ct) value of the *lytA* gene obtained through qPCR. From the cases with known Ct value and exact antibiotic start date, a second-degree polynomial (quadratic) regression model was performed to quantify the relationship between the Ct value of the *lytA* gene (explanatory variable) and the days of antibiotic treatment (response variable). We found that there was a statistically significant relationship between the explanatory variables, Ct value, and (Ct value)^2^, and the response variable, days of antibiotic treatment: F(2, 27) = 26.07, *P* < .0001, index of goodness of fit = 0.6589. Combined, these 2 explanatory variables accounted for 65.9% of the explained variability in days of antibiotic treatment. The regression equation was as follows: 


$$\text{Days of antibiotic treatment }=18.9573-1.65517\times (\text{Ct value})+0.03819\times {\left(\text{Ct value}\right)}^{2}$$

The above fitted model was used to estimate the days between the start of antibiotic treatment and the first CSF sample that was positive for pneumococcus, using the Ct value of the *lytA* gene.

#### Sensitivity and Specificity

Considering that (1) previous studies reported sensitivity of ICT to be 95%–100% and specificity to be 99%–100% for detection of pneumococcal meningitis [9, 10, 13]; (2) ICT detected the largest number of pneumococcal cases in this study and did not miss any culture- or qPCR-positive case; and (3) ICT did not yield false-positive results when compared to culture and qPCR of all bacteria detected in this study, here, all ICT-positive cases were considered confirmed pneumococcal meningitis cases. Sensitivity and specificity of culture and qPCR were calculated using ICT as the standard.

### Ethical Considerations

All specimens used in this study were collected from patients as part of routine care at the discretion of the treating physicians. Written consent was obtained from parents or caregivers of all participants for other aspects of the study, including data collection and the use of specimens and isolates for additional laboratory analysis. All protocols were approved by the Ethical Review Committee of the Bangladesh Institute of Child Health, Dhaka, Bangladesh.

## RESULTS

### Pneumococcal Detection by Culture, ICT, and qPCR

From January 2014 through June 2018, we processed CSF specimens from 4334 suspected meningitis cases, 1883 of which contained ≥10 WBC/μL (Figure 1). Median age of the suspected meningitis cases was 10 months (range, 0–177 months) and that of patients with ≥10 WBC/μL in the CSF specimens was 7 months (range, 0–177 months).

All 1883 specimens were available for bacteriological culture, and 1859 (98.7%) were available in sufficient quantity for ICT. Bacterial growth was noted in 32 of the 1883 specimens, 9 of which were *S. pneumoniae* (Table 1). ICT detected pneumococcal antigen in 207 of 1859 available samples, 9 of which were also culture positive. Of the remaining 1652 ICT-negative and culture-negative cases, 1558 samples (96.1%) were available in sufficient quantity to be tested by multiplex qPCR for pneumococcus, *N. meningitidis,* and *H. influenzae.* No additional pneumococcus was detected, but 36 *N. meningitidis* and 9 *H. influenzae* were recorded (Table 1). Of the 207 total pneumococcus-positive cases, 203 were available in sufficient quantity for testing by qPCR for pneumococcus, which was able to detect 184 (90.6%) of the 203 cases. Finally, 1674 of all 1859 specimens were available for antibiotic assay, and in 1172 (70%) of these specimens, presence of antibiotic was noted.

**Table 1.** Pathogens Detected in the 1883 Cerebrospinal Fluid Specimens Tested in The Study and the Method of Detection

Pathogen Detected	No. Culture Positive	No. Antigen/qPCR Positive, Culture Negative	Total
*Streptococcus pneumoniae*	9	198	207
*Neisseria meningitidis*	3	36	39
*Haemophilus influenza*	0	9	9
*Acinetobacter* spp.	6	NA	6
*Flavobacterium* spp.	7	NA	5
*Escherichia coli*	3	NA	3
*Salmonella* spp.	2	NA	2
*Providentia* spp.	1	NA	1
*Pseudomonas aeruginosa*	1	NA	1
Total	32	243	275

Abbreviations: NA, not applicable (antigen/qPCR tests were done for only *S. pneumoniae*, *N. meningitidis*, and *H. influenzae*); qPCR, quantitative polymerase chain reaction.

### Sensitivity and Specificity

In total, 1761 CSF specimens were tested by culture, ICT, and qPCR simultaneously (Figure 1). Culture detected 9 pneumococcal cases, qPCR detected 185 cases (including 9 culture-positive cases), and ICT detected 207 cases including cases identified by culture and qPCR. Sensitivity of culture in detecting pneumococcus was 4.89% (95% confidence interval [CI], 2.26%–9.08%) and 4.43% (95% CI, 2.05%–8.05%) when qPCR and ICT , respectively, were used as comparators (Table 2). Sensitivity of qPCR assay for the detection pneumococcal DNA was assessed as 100% (95% CI, 66.37%–100%) and 90.64% (95% CI, 85.77%–94.27%) when compared to culture and ICT, respectively (Table 2).

**Table 2.** Sensitivity and Specificity of Bacterial Culture, ICT, and qPCR in the Detection of Pneumococcus

Test Assay	Comparator	Sensitivity, % (95% CI)	Specificity, % (95% CI)
Culture	ICT	4.43 (2.05–8.25)	100 (99.76–100)
qPCR	ICT	90.64 (85.77–94.27)	100 (99.76–100)
ICT	Culture	100 (66.4–100)	100 (99.8–100)
qPCR	Culture	100 (66.4–100)	100 (99.8–100)
Culture	qPCR	4.89 (2.26–9.08)	100 (99.77–100)
ICT	qPCR	100 (98–100)	100 (99.8–100)

Abbreviations: CI, confidence interval; ICT, immunochromatographic test; qPCR, quantitative polymerase chain reaction.

Among the 207 cases of pneumococcal meningitis and 68 nonpneumococcal cases, ICT for pneumococcus was negative for all 68 nonpneumococcal cases detected by culture or qPCR, and qPCR for pneumococcus was negative for the 45 nonpneumococcal cases detected by qPCR, indicating 100% specificity of both ICT and qPCR as pneumococcal diagnostics (Table 2).

### Impact of Antibiotic Exposure on Sensitivity of Pneumococcal Detection by Culture, ICT, and PCR

**Figure 2.**
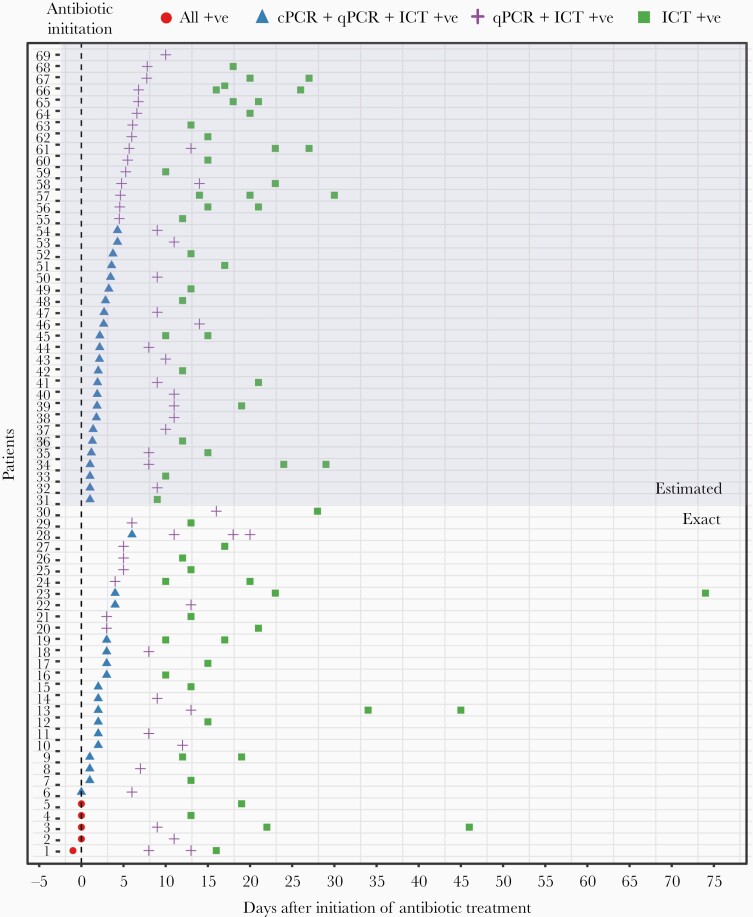
Time of collection and mode of pneumococcal detection of the 165 longitudinal samples from 69 patients with confirmed pneumococcal meningitis. The exact start date of antibiotic treatment was known for patients 1–30. The start date was estimated for patients 31–69 using *lytA* cycle threshold value (see “Methods”). Abbreviations: cPCR, conventional polymerase chain reaction; ICT, immunochromatographic test; qPCR, quantitative polymerase chain reaction.

Among the 207 pneumococcal meningitis cases, longitudinal samples (>1 CSF samples/case, separated by time) were available for 69 cases, collected up to 74 days after initiation of antibiotic treatment (Figure 2). For 49 cases, 2 longitudinal samples were available; for 13 cases, 3 longitudinal samples; and for 7 cases, 4 longitudinal samples were available. In total, 165 samples were available from the 69 cases. Of the 165 samples, 5 were culture positive, 46 were cPCR positive, 101 were qPCR positive, and all 165 were ICT positive.

**Figure 3.**
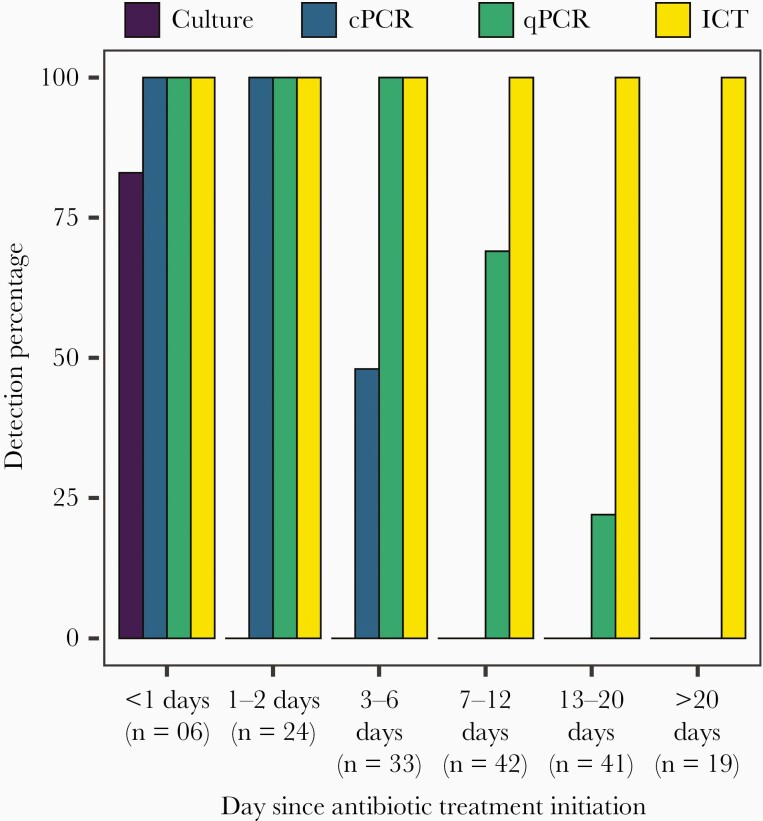
Sensitivity of culture, immunochromatographic test, cPCR, and qPCR for detection of pneumococcus after antibiotic treatment initiation. Abbreviations: cPCR, conventional polymerase chain reaction; ICT, immunochromatographic test; qPCR, quantitative polymerase chain reaction.

The exact date of the start of antimicrobial treatment was known for 30 children; for the remaining 39 children, antibiotic exposure was confirmed by testing for presence of antibiotic in the CSF (see “Methods”), and the treatment start date was estimated based on the Ct value of *lyt*A gene from the qPCR (see methods). Sensitivity of detection of pneumococcal cases by the different diagnostic tests after antibiotic exposure is illustrated in Figure 3.

#### Culture

Only 1 of 165 samples was collected 1 day prior to initiation of antibiotic treatment, and 5 were collected on the day of initiation. Bacteriological culture yielded growth of pneumococcus in 5 of these 6 samples. None of the remaining 159 samples, all of which were collected after antibiotic exposure, were culture positive.

#### PCR

All samples collected within 2 days of antibiotic exposure were positive by cPCR (n = 30). By day 3 of antibiotic treatment, cPCR sensitivity decreased to 95% (39/41; 95% CI, 83.47%–99.40%), and by day 6 to 73% (46/63; 95% CI, 60.35%–83.43%). All 102 CSF samples collected after day 6 of treatment were negative by cPCR. All samples were positive by qPCR up to 8 days after antibiotic initiation (n = 75). Sensitivity of qPCR decreased to 87% (92/105; 95% CI, 79.76%–93.24%) by day 12, and to 69% (101/146; 95% CI, 61.01%–76.55%) by day 20. All 19 samples collected after 20 days of treatment were negative.

#### Immunochromatographic Test

All 165 samples were ICT positive for pneumococcal antigen, including the specimens collected beyond 20 days and up to 74 days.

## Discussion

Use of antibiotics prior to seeking care is widespread in many low- and middle-income countries, making it difficult to detect pathogenic bacteria by traditional bacteriological culture [6, 18]. Previous studies have documented that a large proportion of cases of pneumococcal disease are culture negative [6] in high antibiotic exposure settings, impeding our knowledge on (1) burden and epidemiology, (2) trends and patterns of antimicrobial resistance, and (3) vaccine impact. To close this gap, it is imperative to find appropriate diagnostic(s) and ensure their optimal use. Multiple diagnostics are currently available to detect pneumococcus in the CSF of probable meningitis cases. However, there is an overall lack of consensus about the performance (sensitivity and/or specificity) of the various diagnostics, and laboratories make different choices depending on their setting and access. WHO recommends bacterial culture as the first priority for confirmation and isolation of pneumococcus, but recognizes the low sensitivity due to potential antibiotic usage, and therefore also recommends PCR on all suspected specimens. It also recommends the rapid diagnostic tests, ICT (BinaxNOW) and latex agglutination [15].

In this study, we conducted a head-to-head comparison of contemporary diagnostics: culture, cPCR, qPCR, and ICT after antibiotic exposure. We compared the sensitivity and specificity of culture, ICT, and qPCR in detection of pneumococcus, using CSF specimens collected from 4334 suspected meningitis cases and elucidated the effect of prior antibiotic exposure on the sensitivity of detection of each method using 165 longitudinal CSF specimens collected from 69 patients during their hospital stay. We did not include the commercial latex agglutination kit as it is expensive, has been shown to lack sensitivity, and has a short shelf life [15, 19].

Bacteriological culture of 1883 meningitis cases with ≥10 WBC/μL yielded growth of pneumococcus in 9 samples. Antibiotic assay of 1674 of these CSF specimens showed that 70% of the cases had antibiotic exposure either prior to seeking care at the hospital or before the lumbar puncture was performed. qPCR detected 184 cases and did not miss any of the culture-positive cases. Use of rapid ICT for pneumococcal antigen yielded 207 cases of pneumococcus and captured all qPCR- and culture-positive cases. Overall, sensitivity of culture in detecting pneumococcus was 4.43% (95% CI, 2.05%–8.05%) and that of qPCR was 90.64% (95% CI, 85.77%–94.27%) when compared to ICT, respectively. None of the methods yielded any false-negative results when compared to each other, illustrating 100% specificity of all methods.

Poor yield from CSF culture within 24 hours of prior antibiotic exposure may be explained by high susceptibility to penicillin and cephalosporins of circulating pneumococcal strains in Bangladesh; over 90% of all pneumococcal isolates are estimated to be susceptible to penicillin and 100% to cephalosporins, which is the most commonly consumed over-the-counter antibiotic in Bangladesh [6, 20–22]. Nonpneumococcal pathogens were identified in 68 CSF specimens, and no cross-reactivity of ICT or qPCR for pneumococcus was identified with any of the recorded pathogens. In concordance with previous studies, this confirmed 100% specificity of these diagnostics in precise detection of pneumococcus with the protocols as used in our study for CSF specimens [10, 16, 23].

In addition to assessing the overall performance of the diagnostics, this study also illustrated the change in sensitivity with time following antibiotic exposure. This is especially important in settings like Bangladesh, where patients pay for care out of their pockets and sick people often do not come to receive treatment in facilities unless the symptoms are severe. Sometimes, patients come only several days or weeks after manifestation of symptoms [24, 25]. Longitudinal samples from 69 patients collected at different time points after initiation of antibiotic treatment revealed gradual erosion of sensitivity of culture, cPCR, and qPCR. CSF culture did not yield any positive results starting from the second day of antibiotic exposure and cPCR positivity disappeared after day 6. qPCR is more sensitive and was able to detect pneumococcal DNA in many of the cases until day 20 after initiation of antibiotics, but not beyond that. ICT is the most sensitive of all methods and could detect pneumococcal antigen up to 74 days after antibiotic treatment initiation. It was not possible to pin-point how long pneumococcal antigen remains detectable in CSF by BinaxNow, as ICT still yielded a positive result at the longest time gap of 74 days, despite decreasing WBC count. However, another study showed that BinaxNow can detect pneumococcus for up to 90 days after the initiation of antibiotic therapy [25]. Such prolonged duration of antigen positivity may hinder detection of recurrent infections and may be explained by the fact that BinaxNow recognizes fragmented molecules of the C-polysaccharide, such as teichoic acid, which are produced after antibiotic treatment and can escape phagocytic clearance mechanisms [26, 27].

The findings of this study should be considered within the context of some limitations. First, the exact antibiotic and dose received by the child was not known in many cases due to limitations in caregiver recall. Different antibiotics are likely to have different effects on the sensitivity of the diagnostics with time. Second, the time gap between each lumbar puncture varied between patients as this was completely dependent on the treating physicians, and only performed when deemed absolutely necessary. However, the large number of longitudinal specimens (n = 165) from 69 confirmed pneumococcal-meningitis patients collected over a span of 74 days of antibiotic exposure aided in assessment of changes in sensitivity of the diagnostics with time.

Each of the diagnostics examined have their own limitations, and often we may need more than one diagnostic for a comprehensive clinical and epidemiological assessment. Pneumococcal culture can provide data about serotype, antimicrobial resistance patterns, and additional genomic information. However, with exposure to antibiotics, its sensitivity plummets within hours, limiting its utility to a narrow timeframe. ICT is an excellent tool to detect pneumococcus for clinical purposes and burden studies; it can provide results within minutes, it is the most sensitive tool, and can detect pneumococcus for weeks after antibiotic therapy. However, it cannot provide information about the causative serotype or antibiotic sensitivity; serotype information is crucial for epidemiological surveillance and to understand the impact of PCVs on serotype distribution. In addition, the widely used BinaxNOW test for ICT is expensive and can only detect pneumococcus; other common meningeal pathogens, *H. influenzae* type b, *N. meningitidis,* and group B streptococci, need to be additionally tested using latex agglutination test or PCRs. Hence ICT may be cost prohibitive in some settings. However, as noted above, latex agglutination tests have low sensitivity in comparison to other methods [15, 19] and PCRs are overall less sensitive than ICT, and also cannot generate data on antibiotic sensitivity. qPCR is more sensitive than cPCR, but qPCR is also more expensive with respect to capital equipment, reagents, and technical expertise, and therefore it may be needed to ship specimens to reference laboratories. In contrast to ICT, PCRs can discern serotypes for samples with *lytA* Ct value <35 [28]. Serotype information is crucial for understanding the impact of the current vaccines and to monitor serotype dynamics. We find a tool that can detect pneumococcus with a sensitivity similar to that of BinaxNow and can discern serotypes at the same time. A current immunodiagnostic assay based on multiplex urinary antigen can detect pneumococcal antigen in the urine and can also distinguish between serotypes [29]. A new version of this tool, equipped with identification of 24 serotype-specific antigens along with pneumococcus-specific C-polysaccharides, showed promising results in identification of serotype-specific pneumococci in patients with community-acquired pneumonia [30]. However, this multiplexed immunodiagnostic assay awaits validation in identification and serotyping of pediatric culture-negative pneumococcal meningitis cases in settings with high antibiotic exposure.

## Conclusions

Until we find a diagnostic that is rapid, sensitive, and can identify serotypes and antibiotic susceptibility, we propose that clinical microbiology laboratories or reference laboratories use the diagnostics in the following order: (1) ICT (for real-time diagnosis to inform clinicians and facilitate rational, evidence-based antibiotic treatment based on existing antibiogram data), along with (2) culture (to record the antibiotic sensitivity profile and serotype data, if the organism can be grown) to understand the burden of pneumococcal meningitis in areas where use of prior antibiotic is more common. Finally, qPCR can be used for culture-negative specimens to discern the serotypes for epidemiological data generation; as this will not be required for clinical diagnosis, qPCRs can be performed in batches to lower cost or may be shipped to reference laboratories. If culture, ICT, and qPCR, are conducted optimally, they can aid clinical diagnosis and also elucidate the complete picture of pneumococcal disease in settings with high antibiotic exposures.

## Notes

***Acknowledgments.*** We are grateful to Dr ASM Nawshad, Dr Mohammad Jamal Uddin, and Dr Shampa Saha for clinical and technical support during the meningitis surveillance in Dhaka Shishu Hospital; and Md Shafiqul Islam for technical assistance with building the second degree polynomial regression model.

***Supplement sponsorship.*** This supplement is sponsored by the World Health Organization and the U. S. Centers for Disease Control and Prevention.

***Financial support.*** This work was supported by Gavi, the Vaccine Alliance, through the World Health Organization-supported Invasive Bacterial Vaccine Preventable Diseases Study (grant numbers 201588766, 201233523, 201022732, 200749550, 201686542, 202048618, and 202048971 to Dhaka Shishu Hospital meningitis surveillance) and the Bill and Melinda Gates Foundation (grant number OPP1096930).

***Potential conflicts of interest.*** All authors: No reported conflicts of interest. All authors have submitted the ICMJE Form for Disclosure of Potential Conflicts of Interest. Conflicts that the editors consider relevant to the content of the manuscript have been disclosed.
